# Supplementary material for: A Missense Variant in CASKIN1’s Proline-Rich Region Segregates with Psychosis in a Three-Generation Family
Source: Genes (Basel). 2023 Jan 9;14(1):177. doi: 10.3390/genes14010177 (PMC9859343; doi:10.3390/genes14010177)
Supplement: Supplementary file 1 [file genes-14-00177-s001.zip › genes-2119082-supplementary Table S1-S5, Figure S1-S4.pdf]

genes

Supplementary Materials

A Missense Variant in CASKIN1′s Proline-Rich Region Segregates with Psychosis in a Three-Generation Family

Table S1. Family diagnosis information.

Individual	Age at Diagnosis	Gender	Diagnosis (MINI)	DSM IV Diagnosis (Consensus)	Other Diagnoses
I-2	85	M	No Psychotic Disorder	No Psychotic Disorder	
I-3	81	F	No Psychotic Disorder	No Psychotic Disorder	Anxiety Disorder
I-4	67	F	Psychotic Disorder	Schizophrenia	Postpartum onset
II-3	57	F	No Psychotic Disorder	No Psychotic Disorder	Retinitis Pigmentosa
II-5	55	M	No Psychotic Disorder	No Psychotic Disorder	Retinitis Pigmentosa
II-7	37	F	Psychotic Disorder	Schizophrenia	
III-10	30	M	No Psychotic Disorder	No Psychotic Disorder	
II-11	33	M	Psychotic Disorder	Schizophrenia	Retinitis Pigmentosa
II-13	37	F	Psychotic Disorder	Schizophrenia	Postpartum Onset
III-3	39	M	No Psychotic Disorder	No Psychotic Disorder	
III-8	26	M	No Psychotic Disorder	No Psychotic Disorder	
III-9	27	M	Mood Disorder *with Psychotic Features*	Bipolar Disorder I	
III-11	28	M	No Psychotic Disorder	No Psychotic Disorder	
III-12	15	F	Psychotic Disorder	Schizoaffective Disorder (bipolar type)	

Table S2. Bioinformatic predictions of functionality of variants discovered.

Gene	Variant	Variant locus and allele change	pLI	Missense Z	Presence in databases	VEST	fathmm
RHEBL1	Valine 114 Δ Leucine	Chr12: 49460054 C-->G	0	0.84	N/A in dbSNP, ExAC, gnomAD	0.6	0.98511

CASKIN1	Aspartate 1204 Δ Asparagine	Chr16: 2229759 G-->A	1	1.79	N/A in dbSNP, ExAC, gnomAD	0.025	0.94836

Score descriptions:

- **pLI:** Probability of loss of function intolerance.
- **Missense Z: Z** scores indicate intolerance to missense variation. Z scores larger than 2 indicate intolerance at the 5th percentile.
- **VEST:** Predicts the functional significance of a missense mutation based on the probability that it's pathogenic. Scores near 1 indicate a functional prediction.
- **fathmm:** Predicts the consequences of coding variation – The range is 0-1 values above 0.5 are predicted to be deleterious

All genomic positions are in hg19

Table S3. SCZ and BPD PRS for penetrant and non-penetrant individuals.

ID	Diagnosis	PRS-SZ-threshed p = 0.00115	PRS-BPD threshold p<5e-4
II-3	Non-penetrant	0.00045	-0.00388
III-12	Schizoaffective	0.00063	-0.00447
III-9	Bipolar - I	0.00053	-0.00412
I-3	Non-penetrant	0.00040	-0.00393
I-4	Schizophrenia	0.00067	-0.00479
II-7	Schizophrenia	0.00051	-0.00432
II-13	Schizophrenia	0.00069	-0.00411
II-11	Schizophrenia	0.00053	-0.00411

Table S4. On target and off target primers.

Locus	Forward primer	Reverse primer
Chr16:2179957-2179555	5'-CGTCAAGTTCATCCTGACCG-3'	5'-GAGTAAGGAGGTGGAGCAGG-3'
ChrX: 154468525-154468995	5'-GAGAGCAAGAACCACCCCAA-3'	5'-CTCTCTCTGCACCCCTACCT-3'
Chr16:71168265-71168864	5'-GCAGAGATCACACCATTGCACT-3'	5'-GAACCACAAATTTTGATTGGACAT-3'
Chr1: 146541273-146541827	5'-TAGTCTCCAAATTTTCAGGGCGG-3'	5'-AGTGAGCAGAGATCACACCA-3'

Table S5. sgRNA oligo sequences with Bbs1 sticky ends bolded and ssODN sequence.

Forward Oligo	Reverse Oligo	Forward Oligo
5′-**CACC**GG-TAGGTGCGCCAGGTCGGT-3'	5′-**AAAC**ACCGACCTGGCG-CACCTACC-3'	5′-**CACC**GG-TAGGTGCGCCAGGTCGGT-3'
**ssODN:** 5′-CTTGGGAGAGACAGGCGGCTTGGCCGGCTTCCGGGCTTCGCCCTCGGGCGGGGG-CAATGaGGGTAGGTcCGaCAGGTtGGTGGGCGGGGGTTCGGCAGGCGGGGGCGGTGGAGGCAGCTCCGGAGGCCCAGCCTGCTCCGAGGCCGGTCGGCGGCGCACGG- 3'		

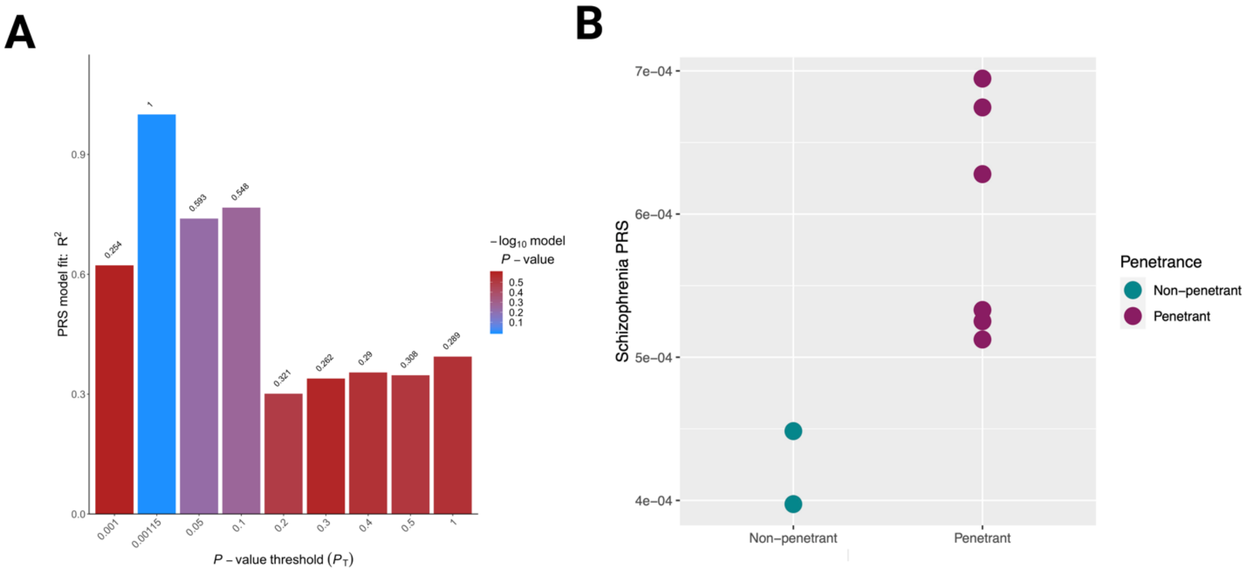

Figure S1. PRSs corelate with the penetrance of CAKSIN1 D1204N. (A) Correlation of score with penetrance at different p-value thresholds. Correlation reaches 1 at a threshold of 0.00115. **(B)** PRS scores of for each individual at the best threshold.

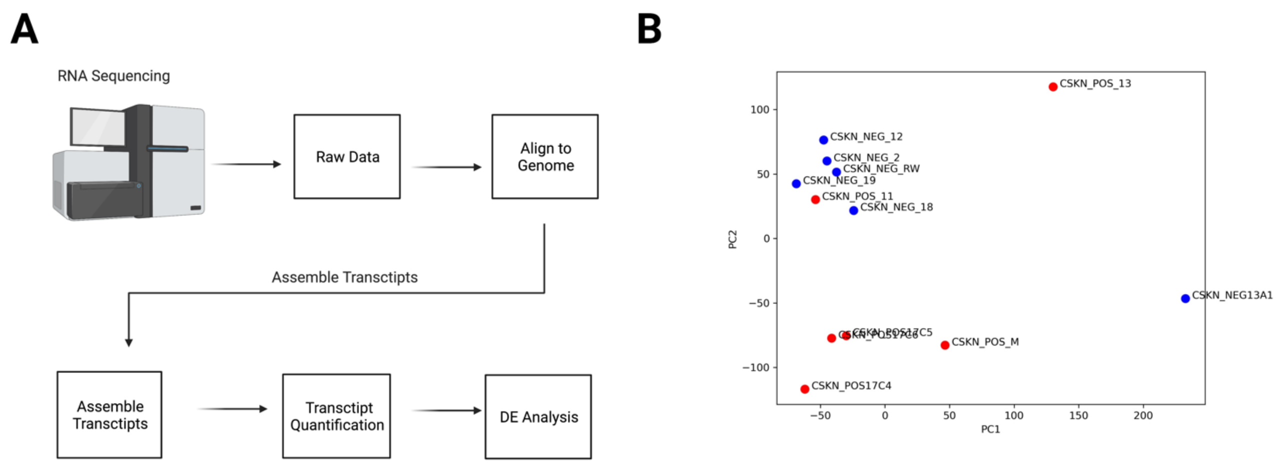

Figure S2. RNA-seq flow and PCA. (A) Flow of RNA-seq analysis. **(B)** Principal Component Analysis (PCA), PC1 and PC2. Each dot is a clone. All red clones are edited. All blue clones are unedited. PC1 separates out 2 clones, one edited and one unedited. These clones were taken out of RNA-seq analysis.

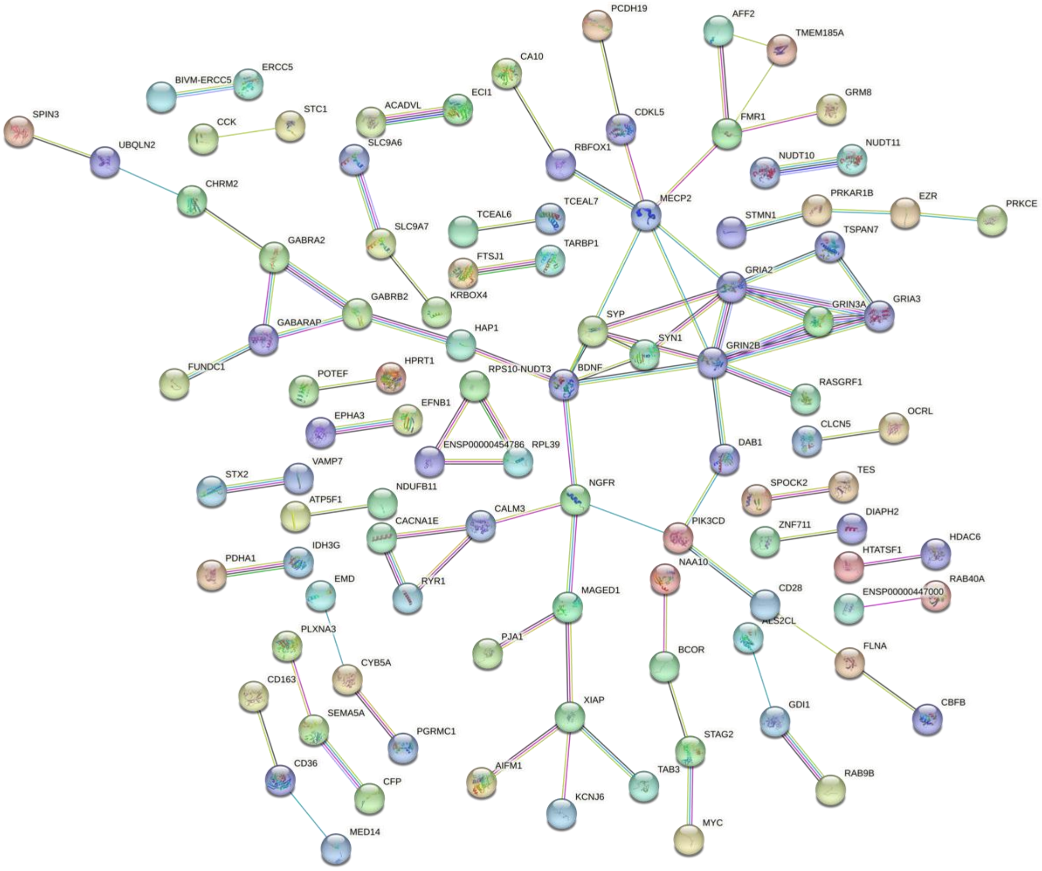

Figure S3. STRING analysis of protein-protein interactions of DEG. Network nodes represent proteins of significantly DEG genes (padj<0.05) that have an interaction with one or more proteins within the same set of genes as represented by the Edges. The minimum required interaction score was 0.7 (high confidence). We found a strong enrichment in PPI with 89 observed edges as compared to the expected 47 for a random set of genes (p-value: 2.5e-08).

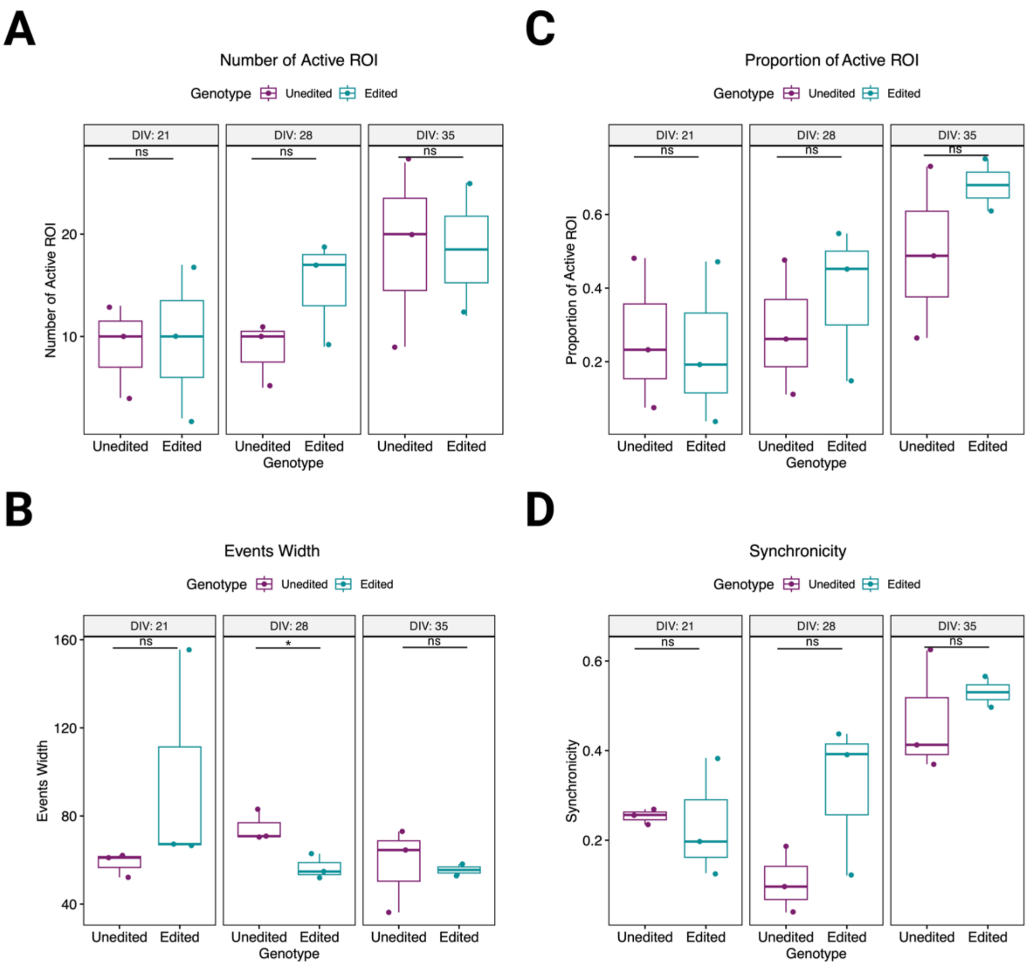

Figure S4. Calcium imaging data. **(A)** Number of active ROIs in edited (teal) and unedited (purple) cells across 3 time points (all p-values are nominal): DIV21(p = 0.903), DIV28 (p = 0.166), and DIV35 (p = 0.986). (B) Events width in edited (teal) and unedited (purple) cells across 3 time points: DIV21(p = 0.327), DIV28 (p = 0.282), and DIV35 (p = 0.850). (C) Proportion of active ROIs in edited (teal) and unedited (purple) cells across 3 time points: DIV21 (p = 0.876), DIV28 (p = 0.569), and DIV35 (p = 0.312). (D) Synchronicity in edited (teal) and unedited (purple) cells across 3 time points: DIV21 (p = 0.839), DIV28 p = 0.156), and DIV35 p =0.528).
